# Catalytic Production of Levulinic Acid (LA) from Actual Biomass

**DOI:** 10.3390/molecules24152760

**Published:** 2019-07-30

**Authors:** Michela Signoretto, Somayeh Taghavi, Elena Ghedini, Federica Menegazzo

**Affiliations:** CATMAT Lab, Department of Molecular Sciences and Nanosystems, Ca’ Foscari University of Venice and INSTM RUVe, via Torino 155, 30172 Venezia Mestre, Italy

**Keywords:** three generations of biomasses, actual biomass transformation, hydrolysis, levulinic acid, catalysts, biorefinery

## Abstract

Catalytic conversion of actual biomass to valuable chemicals is a crucial issue in green chemistry. This review discusses on the recent approach in the levulinic acid (LA) formation from three prominent generations of biomasses. Our paper highlights the impact of the nature of different types of biomass and their complex structure and impurities, different groups of catalyst, solvents, and reaction system, and condition and all related pros and cons for this process.

## 1. Introduction

Among the challenging issues that humans are struggling with in the 21st century, Climate change and Energy security are considered as the most important issues that need to be addressed [1]. With the rapid depletion of fossil fuels, international attempt to raise the use of renewable energy such as biomass has greatly increased [2,3,4]. Various approaches, including thermal, biological, and chemo-catalytic processes have been performed in order to produce fuels and chemicals from biomass, owing to environmental and economic needs [5]. Two different types of processes are used for this application. The first type is thermochemical process in which the entire biomass could be considered as feedstock. These processes mainly include gasification, liquefaction, pyrolysis, and high-pressure supercritical extraction. On the other hand, individual fractions of biomass, including starch, sugars, cellulose, and fatty acids could be separated and transformed by a hydrolysis step (catalyzed by acids or bases), after which there are several processes for transforming each one [6]. Acid-catalyzed hydrolysis has been considered to be a crucial step for chemicals production at a relatively mild temperature (100–250 °C). Furfural, glucose, 5-hydroxymethylfurfural (5-HMF), and levulinic acid (LA) are regarded as the key intermediate platform chemicals [7].

**Figure 1:**
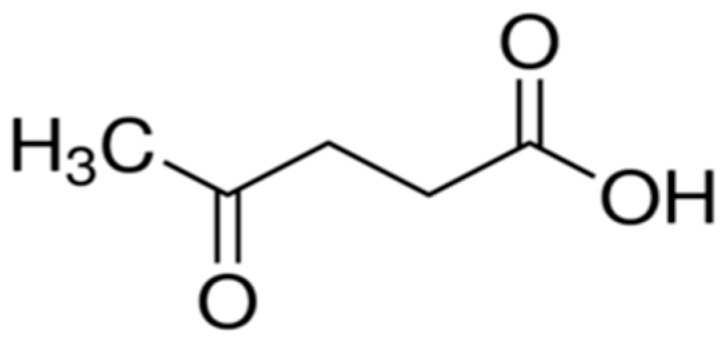
The molecular structure of levulinic acid.

LA is a linear C5-alkyl carbon chain that is known as 4-oxopentanoic acid or gamma ketovaleric acid and also 3-acetylpropionic acid. It is a short chain fatty acid with molecular formula C_5_H_8_O_3_ (see Figure 1) [8]. The Biomass Program of the US Department of Energy in 2004 regarded LA as one of the top 12 most promising bio-based platform chemicals [2].

**Figure 2:**
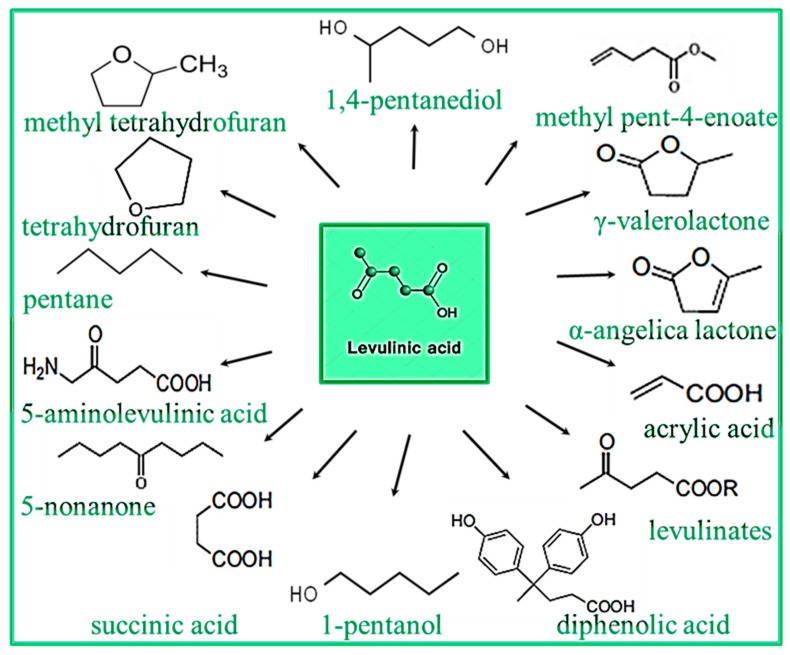
Levulinic acid (LA) derivatives [1,17].

LA acts as a viable chemical bridge between biomass and petroleum processing. Multiple LA derivatives have been suggested for fuel applications, such as γ-valerolactone (GVL), ethyl levulinate (EL), and methyl tetrahydrofuran (MTHF). One of the most important processes is the hydrogenation of LA to γ-valerolactone, and further to liquid alkenes with eight or more carbons. In addition, LA can be used as a as gasoline and biodiesel additives by conversion to a family of valerate esters [9,10,11,12,13,14,15,16,17]. Besides renewable biofuels, LA is also a promising basic chemical for other applications. As shown in Figure 2, LA can be converted to a range of multiple derivatives that have various market applications. The chemicals that are produced from LA are currently used in several industries, such as solvents, resins, chemical intermediates, polymers, electronics, batteries, adsorbents, photography, plasticizers rubber, cosmetic, drug delivery systems, textiles, and pharmaceutical products [7,8,18,19,20,21,22,23,24,25,26,27,28,29].

There are several different feedstocks for synthesis of LA, including raw materials and precursors, such as polysaccharides, monosaccharides, furfural, 5-hydroxymethyl furfural (5-HMF), and renewable resources, such as the most types of biomasses. Polysaccharides, including starch, cellulose, hemicellulose, and chitin, are the main components of biomass. Their hydrolysis could lead to the formation of monosaccharides, such as glucose and fructose [30]. 

**Figure 3:**
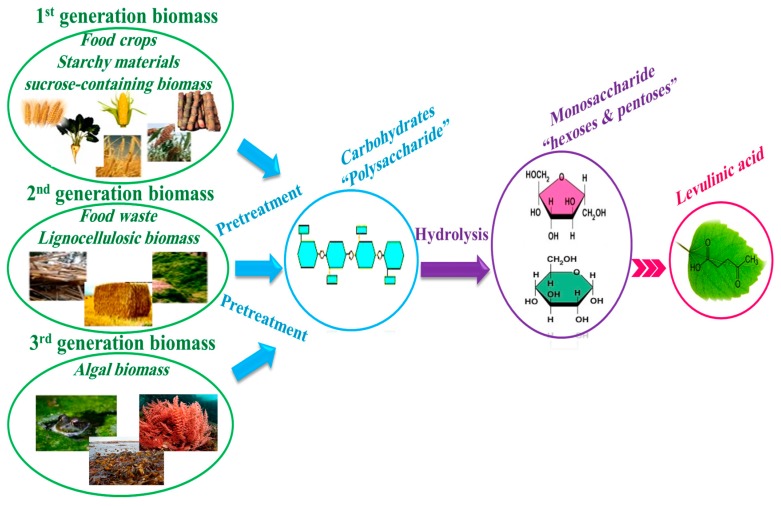
Roadmap of LA production from biomass.

As can be seen in Figure 3, there are three different generations of biomasses:

(i) First generation of biomass comes from food crops such as sugar, starchy crops, vegetable oil, or animal fat.
(ii) Second generation of biomass is non-food crops such as wood, organic waste, food crop waste, and specific biomass crops. Most of biomasses in this generation are considered as the lignocellulosic biomass.
(iii) Third generation of biomass comes from algae.

Therefore, the reaction of LA production from three different generations of biomass is consecutive and usually includes three general steps (see Figure 3):

(i) pretreatment of biomass to extract polysaccharides,
(ii) hydrolysis of polysaccharides into monosaccharides such as hexoses and pentoses, and pentoses,
(iii) conversion of monosaccharides to LA during several steps [31].

The pretreatment of biomass is a compulsory and significant step in its transformation to LA in order to have a great LA yield and reaction rates since the structure of raw biomass is very complex. Moreover, the type and severity of pretreatment could be of crucial importance, according to the complexity of the raw starting biomass [32]. The next important route is several steps acid catalyzed reaction for formation of LA from different types of C_5_ and C_6_ sugars, which can also drive from the hydrolysis of polysaccharides or more complex carbohydrates extracted from pretreatment of raw biomass, such as cellulose, hemicellulose or starch. For these reactions the acidity of the catalyst plays a crucial role in which both Lewis and Brønsted acid sites in catalyst and a great balance of them are highly needed.

Although several researches have been undertaken on the tailored acid catalyzed hydrolysis of biomass, and this interest continues to rise in the recent years, few review papers have been published regarding the synthesis of LA from real biomass, especially focusing on using three different generation of biomasses as a feedstock, including starchy, lignosellulosic, and marine biomasses [4,33,34]. Thus, in this review, we focus on different biomass categories and using catalysts in the real biomass conversion and the production of one of the valuable chemical, named LA, based on recent years’ studies.

## 2. LA Production from First Generation of Biomass: Starchy and Sugary Biomass

**Figure 4:**
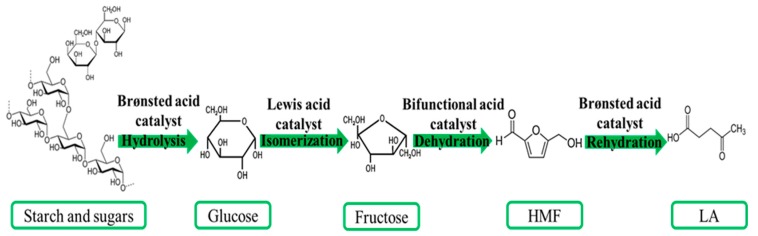
Overview of LA production from first generation biomass.

First generation of biomass consisting starchy and sugary biomass can be converted to LA during several steps (see Figure 4): (1) the hydrolysis of starch or sugar as a polysaccharide to glucose catalyzed by a Brønsted acid; (2) isomerization of glucose to fructose by using a Lewis acid; (3) dehydration of fructose to 5-HMF catalyzed by bifunctional acid; and, (4) rehydration of 5-HMF to LA catalyzed by Brønsted acid. It was demonstrated that the conversion pathway of glucose to 5-HMF depends on the type of the catalyst. It has been established that fructose can only be formed as an intermediate when Lewis acid catalysts are used in this reaction considering that not all Lewis acid sites are active for glucose isomerization, but they simultaneously act as a catalyst for side reaction or soluble polymers and insoluble humins. On the other hand, if a strong Brønsted acid has catalyzed the reaction, glucose dehydration follows a route with 3-deoxy-glucosone as an intermediate [35,36,37]. Therefore, there are several reaction networks happening through transformation of biomass to LA: (1) isomerization of glucose to fructose, which dehydrates to HMF and rehydrates to LA and formic acid; (2) direct dehydration of glucose to HMF; (3) direct dehydration of glucose to furfuryl alcohol which converts to LA and byproducts; and, (4) side reaction of glucose, fructose, HMF, and furfuryl alcohol, which leads to the production of byproducts, especially tarry humins and char-byproducts. Different types of techniques are suggested to prevent side reactions by controlling the process significant parameters, such as acidity, pressure, temperature, pressure, reaction time, and conducting in situ extraction since the structure of biomass is very complex. Additionally, all these side reactions are probable in transformation of second and third generations of biomass. 

As shown in Table 1, in the last few years, there are few researches that focus on and using first generation biomass in order to produce biochemical. In all of these works, homogenous catalysts were used for hydrolysis process. 

In 2016, a paper reported LA production from three different corn starches, including normal corn starch, high-amylose corn starch, and waxy corn starch while using both microwave and conventional oil bath heating [38]. For both methods, water was used as the solvent and HCl was preferred as the catalyst rather than other mineral acid catalysts because the authors claimed that HCl can be more effective for LA production. In addition, temperatures of 135, 150, 165, and 180 °C were set and a hold time of 0, 5, 10, or 15 min. was applied to the runs. In microwave heating method, the LA yield for waxy corn starch in the shorter reaction time (0 and 5 min.) were the highest one while there were a very slight differences at higher reaction times and temperatures concluding that the lower equivalent temperatures and shorter reaction times could be considered for microwave heating. Anyway, the maximum yield of LA was around 53−55% for all substrates and for both heating media at optimum temperature of 165 °C and time of 15 min. Microwave- assisted heating can be used as a more effective heating method than traditional ones. The use of microwave heating causes a homogeneous heat transfer into the biomass feedstock by dipole rotation and ionic conduction using less energy when compared to the traditional heating method. Hence, microwave reactor results a significant time and energy saving [39].

In 2002, kernel sorghum grain was transferred to LA by using aqueous solution of H_2_SO_4_ as the catalyst. The reaction was performed in several conditions, including different H_2_SO_4_ concentration, sorghum flour loading, and temperature. The yield of LA was increased with the decrease of the sorghum flour loading and increase of temperature and H_2_SO_4_ concentration. The maximum LA yield of 33% was attained at 200 °C, 8% H_2_SO_4_ concentration, and 10% flour loading [40].

Sugar Cane Molasses is a biomass very rich in sucrose, which can be easily hydrolyzed to hexoses, such glucose and fructose. Additionally, Sugar Cane Molasses itself has a slight amount of glucose and fructose in the structure. A recent study [41] proposed a superimposed reaction, in which the LA solution formed from the hexose hydrolysis reaction could be further used as the solvent for additional hexose hydrolysis to produce more LA. First, the optimum condition of reaction was selected as 180 °C for 180 min. H_2_SO_4_ concentration was one of the critical factors that affected the product distribution. Therefore, a relatively high concentration of H_2_SO_4_ (0.2 M) was used as the catalyst for the following works. An average yield of 30 and 24% LA was obtained in the third and fifth superimposed reactions, respectively. In addition, a similar biomass (sugar beet molasses) was used in another study for LA formation. The authors suggested an acidic cation exchange resin (Amberlyst-36TM) as the heterogeneous catalyst for this reaction. However, a pretreatment step was performed due to the rapid deactivation of the catalyst for the presence of non-sugar components, such as cations, proteins, and alkaline compounds in the biomass. In order to remove the impurities of the biomass, it was transferred from a column packed with the resin pellets which was also recovered several times under ambient conditions for repeated pretreatment. The highest LA yield of 78 mol% was produced in the optimum reaction condition of 0.2 g/mL catalyst dosage, 140 °C and 180 min. [42]. 

As can be seen in the Table 1, in all of the works, mostly homogeneous catalysts were used for LA production from first generation of biomass. Although these types of catalyst exhibit favorable yields with reasonable cost, they cannot be considered as promising catalysts due to limited recyclability, reactor corrosion, and waste generation [43,44,45]. These drawbacks can be overcome by using appropriate heterogeneous catalysts instead of homogeneous ones. Therefore, using a cost-effective and environment-friendly catalyst with a reasonable mass transfer, activity, and stability is required. 

However, producing fuels and chemicals from edible biomass has become a disputable topic since there are millions people in the world struggling with the challenge of accessing to the sufficient food. In addition, using first generation biomass leads to several challenges, such as negative impact on food security, violations of people’s rights and livelihoods by large-scale land acquisition for sourcing biomass, biodiversity and water preservation, and negative impact of biomass plants on local air quality through processing and transport emissions and on the aesthetics of the local landscape. Hence, all of these different challenges regarding using edible biomass cause a focus on developing second generation technologies to produce fuels and chemicals from food waste or nonedible feedstocks [46,47,48,49].

## 3. LA Production from Second Generation of Biomass: Food Waste and Lignocellulosic Biomass

**Figure 5:**
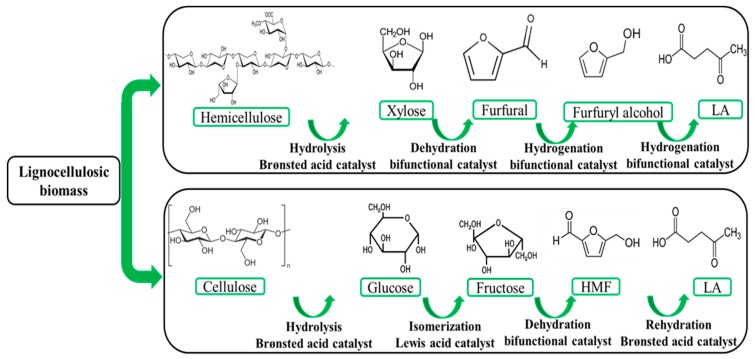
Overview of LA production from second generation biomass.

Second generation or lignocellulosic biomass consisting hemicellulose and cellulose is one of the most plentiful renewable resources. As can be seen in Figure 5, cellulose can be hydrolysed into glucose by using Brønsted acid as the catalyst that further dehydrates into 5-HMF and then rehydrates into LA catalyzed by Brønsted acid catalyst on the course of lignocellulosic biomass conversion. Besides, hemicellulose can be hydrolyzed into xylose by using Brønsted acid, followed by dehydration of xylose to furfural and hydrogenation of furfural towards furfuryl alcohol and ethyl levulinate and then LA over bifunctional catalysts [10,13,14,16,17,50,51,52]. Lignin is another organic component of lignocelluloses that is linked with hydrogen, chemical, and covalent linkage to cellulose and hemicellulose and packs them densely. Most of the lignin remains as the solid residue through LA formation, and only a slight amount of lignin dissolves in the solution. Lignin can be converted into humins in acidic reaction condition and reduce the yield of LA [53]. Pretreatment of actual biomass is one of the prominent methods for isolating cellulose and hemicellulose of biomass from lignin before the hydrolysis. Dilute acid pretreatment is one of the most common methods for biochemical production [54,55].

Several works reported synthesis of LA from different types of straw (rice straw, cotton straw, barley straw, Paddy Straw) by using ionic liquid, homogenous, and heterogeneous catalysts. In 2018, by using Acidic ionic liquid (IL) [C_3_SO_3_Hmim]HSO_4_ as a catalyst, at 180 °C and after 30 min. of one pot reaction, yield of 96 mol% (21 wt%) was obtained from rice straw. It was proven that acidity and hydrogen bonding ability of anions are viable to the yield of LA. In addition, the catalyst was reused over five cycles without any loss of activity [56]. Another work reported an improvement rice straw accessibility to a solid superacid S_2_O_8_^2−^/ZrO_2_–SiO_2_–Sm_2_O_3_ catalyst by using enzymatic pretreatment and phydroxyanisole inhibitor reduced the side reactions during reaction processes, which led to LA yield of 25 wt.% under the optimal condition [57,58]. Moreover, Ga salt of molybdophosphoric acid, GaHPMo, was found to be a possible alternative to the conventional mineral acids used for the production of LA yield of 46 wt.% from rice straw. GaHPMo exhibited superior catalytic performance in terms of activity for glucose conversion and selectivity for LA production relative to the parent HPMo [59]. As a homogeneous catalyst, HCl was used as a catalyst during the hydrolysis reaction of straw, because of its low cost and effectiveness. In 2016, a group of authors suggested several chemicals reagents, such as H_2_SO_4_, NaOH, NaClO_2_, and NaClO for the thermo-chemical pretreatment of rice straw. Subsequently, they performed a post-pretreatment of rice straw fibers for LA formation in a co-solvent reactor system consisting of aqueous HCl, tetrahydrofuran (THF), and Dimethyl sulfoxide (DMSO). The highest LA yield was 21% by using H_2_SO_4_ as the pretreatment reagent [60]. In 2018, the author provided LA production from rice straw while using a co-solvent biphasic reactor system and HCl and dichloromethane organic solvent. The author claimed that, beside HCl, the acidic product could catalyze the hydrolysis reaction (auto-catalysis). The optimum yield of LA achieved 15% wt. in this work [61]. Another study reported that a low concentration of LA does not provide enough H^+^ for hydrolysis of the cellulose and, hence, cause a low reaction rate. On the other hand, too high concentrations lead side reactions, which may negatively affect the rate of hydrolysis of the cellulose to LA. Therefore, by using HCl with the concentration of 4.45% in optimum condition of reaction, the maximum yield of 24% LA was produced from paddy straw [62]. In addition, in some works, a dilute concentration of H_2_SO_4_ was used as a catalyst during hydrolysis reaction. In one of the study, a 9.5% yield of LA was produced from cotton straw in two-step hydrolysis in the optimum hydrolysis condition [63], while in another study 0.03 g/L of LA was produced from barley straw in which the H_2_SO_4_ acid improves dissolving hemicellulose fraction of the straw [64].

In 2017, several types of lignocellulosic biomass (palm oil frond, rubber wood, bamboo and rice husk) were converted to LA in one-pot reaction. Dicationic ionic liquids, containing 1,1-Bis(3-methylimidazolium-1-yl) butylene ([C_4_(Mim)_2_]) cation with counter anions [(2HSO_4_)(H_2_SO_4_)_0_], [(2HSO_4_)(H_2_SO_4_)_2_] and [(2HSO_4_)(H_2_SO_4_)_4_] were used in different hydrolysis conditions for all types of biomass. However, under optimum experimental condition (100 °C, 60 min.), [C_4_(Mim)_2_][(2HSO_4_)(H_2_SO_4_)_4_] gave a higher yield of LA up to 47.52 from bamboo biomass. Thus, the authors concluded that the yield of LA increases with the increase of the number of HSO_4_ in anion, which leads to an increase in the acidity and decrease in viscosity of IL [65]. 

Wood is one of the most plentiful biomass resources in the world, consisting of around 40–45% cellulose and about 20–30% of hemicellulose; both can be readily hydrolyzed to monomeric sugars and then sugars are appropriate compounds for both energy and chemical production [66]. There are several papers that investigate different woody biomass (eucalyptus wood, red pine wood, poplar branches, grapevine pruning, pine sawdust, aspen, fir, birch wood) hydrolysis with various homogeneous catalysts (HCl, H_2_SO_4_, and H_3_PO_4_) in the several reaction conditions. In 2015, a work proposed the use of methanol as a solvent for LA synthesis from eucalyptus wood chips and by using H_2_SO_4_ catalyst. 90 vol % methanol solution as the solvent demonstrated great performance in the inhibition of humins production from glucose by a quick reaction with glucose and convert it into LA and methyl levulinate (MLA). Therefore, 66 mol% of LA and MLA was produced in best reaction condition at 180 °C and 90 min. [25]. In 2016, the same authors used the same biomass (eucalyptus wood) and catalyst (H_2_SO_4_) to produce LA. First, for xylose recovery from hemicellulose, a pretreatment in the mild conditions was done. Subsequently, the pretreated solution was reused in optimum condition of 170 °C and 300 min. to produce 105 g/L of LA. The authors justified that high concentration of old LA in reaction solution leads to some interaction with other intermediates and byproducts reducing the production of LA from glucose [53]. The conversion of *Pinus pinaster* wood into LA while using two consecutive treatments with hot, compressed water was also reported. Water-solubles and hemicellulose solubilization were removed in the first and second step pretreatment, respectively. The pretreated liquid was mixed with H_2_SO_4_ as the catalyst and hydrolyzed at different acid concentrations, temperatures, and reaction times. 66% yield of LA was produced in the optimum reaction condition of 135 °C and 3600 min. [67]. In 2012, another work designed two-step acid-catalytic conversion of hybrid poplar wood chips into LA in order to inhibit humins production from pentose fraction in biomass in severe acid conditions. The reaction was started with a mild acid extraction to remove most of the pentoses and then followed by second harsher step by using a high concentration of H_2_SO_4_ as a catalyst to convert the first step extracted solids to LA. A maximum molar yield of 17.5 wt % based of the initial biomass was produced in the best reaction condition [68]. Kuznetsov et al., in 2013, studied the LA production from different kinds of wood (aspen, pine, fir, birch) using steam conversion of wood impregnated with H_2_SO_4_ (5 wt %) as a catalyst at 220 °C for 120 min. The highest yield of 24% LA from wood was attained in all types of wood. Additionally, the authors mentioned that the ability of inorganic acids as a catalyst to hydrolyze carbohydrate into LA could be considered in the following activity order: HCl > H_2_SO_4_ > H_3_PO_4_ [69]. 

There are several studies focusing on corn stalk, corn cob residue, and corn stover as a biomass for LA production. In 2015, FeCl_3_ solution was proven as a catalyst for the synthesis of LA from corn stalk. FeCl_3_ solution played a positive role in LA production in high temperature and concentration. The highest LA yield was produced at 48.7% under the optimum condition of 230 °C and 10 min. with 0.5 mol/L FeCl_3_ solution [70]. Another study reported a high yield of LA (70%) from corn stover. First, biomass was loaded to an enzymatic saccharification step for glucose production and also isomerization of glucose to fructose. Subsequently, fructose conversion to LA was performed in an effective mixture of acidic ionic liquid [BMIMSO_3_H]HSO_4_ and DI water without any extra need for an acid catalyst [71]. A new fed-batch process for increasing the concentration of LA produced from corncob residues in H_2_SO_4_ solution was recently designed by Liang et al. The fed-batch process (seven stage) was performed after a pretreatment in 3 wt. % H_2_SO_4_ solution to remove the hemicellulose from corncobs. The mass concentration of LA was raised from 23.6 g/L to 107.9 g/L after first and seventh hydrolysis, respectively. The authors claimed that this new process led to a decrease in the amount of acid catalyst in the reaction and a reduction of energy consumption. Additionally, they found that LA yield during the fed-batch process was dropped, owing to the polymerization of the 5-HMF and the glucose to soluble humins analogues [72]. An efficient homogenous catalyst was used for the production of LA from corncob residue by Zhao et al. Using SnCl_4_ as the catalyst was led to 64.6 mol% yield of LA at 180 °C after 60 min. The authors demonstrated that, after hydrolysis of SnCl_4_ catalyst in water, stannic oxide, H^+^, and Cl^−^ could be produced. Therefore, cellulose hydrolysis was catalyzed by Cl^−^ and H^+^, fructose dehydration and 5-HMF decomposition were improved by H^+^ as Brønsted acid, both glucose-to-fructose isomerization and fructose consumption yielding undesirable polymers were catalyzed by Sn(IV) species when considering that it can have some negative impact on cellulose hydrolysis [73]. Furthermore, corncob was used as the raw biomass for LA production that was catalyzed with acid modified zeolite as a heterogeneous catalyst in subcritical condition. The natural zeolite was modified while using a different ratio of HCl solution. By increasing the ratio of HCl in zeolite, the H^+^ ion increased in the surface of zeolite and subsequently in reaction solution improving the hydrolysis of cellulose and hemicellulose to produce monomeric sugars. Additionally, acidic condition and high temperature boosted the dehydration of monomeric sugars to LA. The highest yield of LA was 262 mg/g obtained at 200 °C during a reaction time of 60 min., and zeolite to acid ratio of 1:15. In addition, this catalyst indicated a gradual deactivation and an acceptable stability and reusability after five cycles [74]. 

Sugarcane bagasse, in several countries, is produced in a vast amount as by-products of agro-industrial production. In addition, this type of biomass is potentially prone in LA production with a relatively lower market price. Two studies proposed the use of sugarcane bagasse as a feedstock for LA production. In 2013, 63% LA was directly produced by using 0.55 M H_2_SO_4_ as the catalyst in the optimum condition of 150 °C and 360 min. [75]. In 2017, first, a pretreatment of Sugarcane bagasse with H_2_SO_4_ acid at 120 °C and delignification with NaOH alkali at 80 °C were performed. Afterwards, an acid hydrolysis carried out in different condition. The highest LA yield of 55.00 ± 0.36% was produced at 170 °C and 75 min. with the presence of H_2_SO_4_ as the reaction catalyst [76].

Jeong et al., demonstrated the application of *Quercus mongolica* as a biomass for LA production. They suggested a pretreatment by using H_2_SO_4_ dilute acid at 150 °C in 10 min. After this acid-catalyzed pretreatment, the liquid hydrolysate was rich of the hemicellulosic C_5_ sugars, whereas the solid fraction contained the C_6_ sugars. In 2017, they used the solid fraction for second step acid-catalyzed treatment by using H_2_SO_4_ as the homogeneous catalyst. The highest LA yield of 16.5% (g/100 g biomass) was produced in the optimum condition [77]. On the other hand, in 2018, they used the liquid hydrolysate for catalytic conversion of C_5_ sugar to LA by using the heterogeneous catalyst of alkaline-treated zeolites Y (commercial). The maximum yield of 4.6% LA was produced at 190 °C and 180 min. [78].

In 2012, a study proposed the use of a hybrid catalyst made of HY zeolite and CrCl_3_ in one pot conversion of empty fruit bunch and kenaf to LA. The author claimed that the catalytic reaction of the catalysts was predominantly influenced by the type of acid sites (Lewis acid), acid sites density, pore size, and shape selectivity. Hydrolysis of empty fruit bunch and kenaf at optimum temperature of 145 °C and reaction time of 146 min. produced 53% and 66% of the LA, respectively. This high yield of LA was justified by the suitable acidity of catalyst, the sufficient microspores and mesoporous diameter to decrease some side reactions (fragmentation and polymerization), and proper shape selectivity, which leads to trap 5-HMF (intermediate product of glucose) within the cage and rehydrate by acid sites to form LA [79]. In addition, using metal halide (CrCl_3_) beside zeolite demonstrated great catalytic reactivity by improving the LA yield. CrCl_3_ as a Lewis acid site promoted the glucose isomerization, whereas both Brønsted and Lewis acid sites of CrCl_3_ and zeolite improved the dehydration/rehydration reaction to LA. 

In 2017, the oil palm fronds were transferred into LA while using an acidic ionic liquid 1-sulfonic acid-3-methyl imidazolium tetrachloroferrate ([SMIM][FeCl_4_]) as the catalyst. Two important properties of ionic liquid for biomass dissolution and high acidity to catalyze the overall reaction made [SMIM][FeCl_4_] a potential catalyst for direct conversion of oil palm fronds into LA. The optimum yield of 25% was produced at 154.5 °C during 3.7 h of reaction time [80].

In 2017, Tiong et al., proposed a heterogeneous catalyst that was made of indium trichloride as the Lewis acidic site and a noncorrosive ionic liquid, 1-methylimidazolium hydrogen sulfate as the Brønsted acidic site to produce LA from oil palm empty fruit bunch and mesocarp fiber biomass. The authors suggested that the concentration of catalyst is one of the most important factors in the depolarization of biomass, because the excess loading of catalyst causes cross polymerization, acceleration of the reducing sugars degradation, and the production of black insoluble charred materials, the so-called “humins”. Furthermore, the presence of InCl_3_ as the Lewis acid helped the production of desirable product by promotion of glucose to fructose isomerization. The best result was 12% and 13% yield of LA conducted at ionic liquids-to-biomass ratio of 5:1 (*w*/*w*), 0.15 mmol InCl_3_, and temperature of 160 °C for 300 min. from oil palm empty fruit bunch and mesocarp fiber, respectively [81]. In 2019, the same authors used the same catalyst and biomass feedstock in order to optimize the operation condition by the response surface methodology approach. The best result, including LA yield of almost 18%, was obtained at 177 °C in 288 min. with 0.15 mmol InCl_3_ in ionic liquids-to-biomass ratio of 6.6:1 (*w*/*w*) from both oil palm empty fruit bunch and mesocarp fiber biomass [82]. 

It has been reported that giant reed as a suitable biomass in the Mediterranean area growing under extreme conditions can be directly convert to LA with a maximum yield of 23%. First, when pre-treatment of the biomass was needed, it was performed at a lower temperature for 120 min. Subsequently, the main hydrothermal conversion was accomplished by using HCl as an appropriate homogeneous acid catalyst. The authors believed that HCl could have a great performance, even at low concentration [83]. In 2015, the same authors tried to optimize the reaction condition by using microwave heating method and also the diluted acid approach. Eventually, the maximum LA yield of 21% was obtained at 180 °C for 20 min. [84].

The microwave heating process was also used for the conversion of two types of biomass, including carbohydrate-rich potato peel waste and sporocarps of the fungus *Cortinarius armillatus* to LA. The reaction was performed in the presence of both Brønsted acid (H_2_SO_4_) and Lewis acid (CrCl_3_·6H_2_O or AlCl_3_·6H_2_O) catalysts. The authors reported that the process was dependent on the time, temperature, H_2_SO_4_, and Lewis acid concentrations. The maximum LA yield of 49% and 62% was achieved in the optimum reaction conditions of 180 °C, 15 min. and 180 °C, 40 min. from potato peel waste and *Cortinarius armillatus,* respectively [85]. Since the solid heterogeneous catalyst has polarity, it can interact with the microwave field and be heated quickly beside the liquid homogeneous catalyst. Thus, the microwave method can be efficient for the reaction due to great thermal influence.

In 2013, bamboo shoot shell was transferred into LA that was catalyzed with ionic liquid [C_4_mim]HSO_4._ Although this ionic liquid can be considered to be an effective and environmentally friendly catalyst, when considering their high cost, the authors suggested further investigation of ionic liquids for this application. After optimizing the reaction condition, the best LA yield of 71 mol% was obtained at 145 °C for 104 min. [86]. 

HCl and H_2_SO_4_ are the mineral acid catalysts that were used for the conversion of biomass to LA in the most of the works, as it is obvious from Table 2. Using these homogenous catalysts that are as the common and traditional catalysts for decades allowing for reasonable yield to LA, cost, and easier accessibility. The reactivity of mineral catalysts is related to several prominent factors, such as the strength and concentration of the catalyst, nature and concentration of biomass, and reaction condition, such as time and temperature. 

In addition, metal salts, such as different metal chlorides, demonstrated remarkable catalytic activity with higher yield of LA. In this group of catalysts, metal cations can act as the Lewis acid site and intrinsic Brønsted acidity derives from their hydrolysis. 

In fact, limited mass transfer of solid-insoluble-substrate/solid-catalyst system causes trouble in using heterogeneous catalysts. Owing to this problem, solid phase catalyst can be more appropriate for water-soluble carbohydrates. There are some few researches that focus on using heterogonous catalysts, especially zeolite with mixed Brønsted and Lewis acid centers in which the results showed an acceptable performance of this group of catalyst. 

Moreover, most of the work was performed under an initial and external pressure of an inert gas, such as nitrogen, and the reaction was done in subcritical water. Therefore, the higher pressure of the reaction was produced from high-pressure steam. Water can be a safe and ecofriendly solvent with high thermal conductivity, which can produce ionic product in high temperature and boost the reactivity of the homogeneous catalyst in biomass conversion [87]. However, polysaccharides and biomasses are insoluble in water. The hydrolysis of cellulose to glucose under mild conditions could be a heterogeneous reaction and by using homogeneous catalyst mass transfer is reasonable and the proton of the catalyst can penetrate into the matrix of cellulose [57]. On the other hand, there is limited contact between solid biomass feedstock and solid heterogeneous catalyst while using water as the solvent, and the proton of solid catalyst cannot be widely dispersed in the water solution. To increase the limited mass transfer, using organic solvents, such as THF, DMSO, MIBK, ionic liquids, and biphasic systems (e.g., aqueous/organic solvent, ionic liquids/aqueous) can be efficient [88,89,90,91]. Selecting a suitable organic solvent according to the nature of substrate and catalyst is of crucial importance. Some significant factors, such as solubility of feedstock in solvent, having similar polarity of the solvent and feedstock, impact of solvent on selectivity of the desired product, possible recovery and recycling, its expense, and environmental effects must be taken into account. 

Some authors used ionic liquid as both solvent and catalyst and the LA yield was low in most cases, depending on the type and reactivity of the ionic liquid. Although this group of solvents attached a great attention due to tunable chemical and physical properties, they demonstrate prominent disadvantages, such as high cost, reactor corrosion, high viscosity and lower mass transfer, and hard recovery by distillation method due to low vapor pressure, which need to be strongly considered. Thus, ionic liquids are not completely environment-friendly and have limitation in using it at the industry level [54,55].

## 4. LA Production from Third Generation of Biomass: Algal Biomass

Recent studies in biofuel and biochemical generation have demonstrated the great potential of macroalgae (seaweed) as the third generation of biomass on extracting high value added chemicals [92]. Macroalgae can be considered as a prominent source of some viable compositions, such as alginates, ulyan, agar, fucoidan, etc. [93,94]. Therefore, polysaccharides are one of the original compositions of three groups of brown, red, and green macroalgae [95]. Using macroalgae as a feedstock for valuable chemicals production has some advantages than second generation biomass consisting no presence of lignin in the structure, high carbohydrate content, and very fast growth rate with the consumption of huge amount of CO_2_ [96].

**Figure 6:**
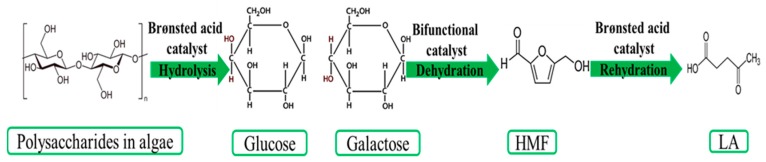
Overview of LA production from third generation biomass.

The brown seaweeds have some types of carbohydrates consisting of laminaran, mannitol, fucoidan, cellulose, and alginates. Agar, cellulose, xylene, mannan, and carrageenan are the saccharides that are presented in the red seaweed cell wall, whereas the green seaweed cell wall is made up of cellulose, mannose, and xylene [97]. As can be seen in Figure 6, different polysaccharides in macroalgae can be hydrolyzed into monosaccharides, such as glucose, galactose, and xylose by using Brønsted acid as the catalyst. Glucose and galactose are prone to follow the process of dehydration into 5-HMF while using bifunctional catalyst and then rehydration into LA using Brønsted acid catalyst. Furthermore, xylose can be dehydrated into furfural and then hydrogenated into furfuryl alcohol and ethyl levulinate and then LA catalyzed by bifunctional catalysts [4,98]. In addition, algal biomass has a considerable amount of inorganic salts in the structure, which can act as a contamination and reduce LA yield. Thus, dilute acid pretreatment of this group of biomass is highly recommended, which can lead to the formation of acid salts and remove the metal ions from the biomass body to aqueous solution.

In 2019, an interesting study has proposed a co-production of LA and hydrochar from red seaweed (*Gracilaria lemaneiformis*), with high potential for economic viability. The authors selected diluted H_2_SO_4_ as the acid catalyst, because of it being common, effective, and cheap. In addition, it is important to consider the calcium naturally present in biomass because a precipitation reaction between the sulphate ions of the catalyst and calcium might happen. They obtained best LA yield of 16.3 wt% through microwave treatment under the conditions of 180 °C, 20 min., 0.2M H_2_SO_4_, and 5% (*w*/*v*) of biomass loading [99]. 

*Gracilaria fisheri* and *Gracilaria. Tenuistipitata* as red seaweeds were also transformed into LA after pretreatment at different concentrations of H_2_SO_4_. This work was conducted to examine the pretreatment conditions to boost the production of fermentable sugars and by-products from these biomasses. The catalytic efficiency for both biomasses was higher when the hydrolysis time was 150 min. and at the same time for *G. fisheri* was higher in comparison with that of *G. tenuistipitata*. The best LA concentration of 3.66 g L^−1^ and 6.12 g L^−1^ was produced in optimum acid concentration of 1 M H_2_SO_4_ with a reaction time of 150 min. at 95 °C for *G. fisheri* and *G. tenuistipitata,* respectively [100]. 

In 2015, a study that focused on optimization of reaction condition for conversion of red-algae *Gracilaria verrucosa* to sugars (glucose, galactose), LA, and 5-HMF by the acidic hydrolysis process. The author reported that LA are prone to being produced at a higher reaction temperature, a higher H_2_SO_4_ catalyst concentration, and a longer reaction time than glucose, galactose or 5-HMF. The best yield of LA was almost 19 wt% at optimum condition of 180.9 °C, 2.85% acid concentration and 50 min. [101]. In 2018, anothers work reported LA production from the same biomass, but using different type of catalyst. MSA (methanesulfonic acid) was applied as a catalyst to this thermochemical reaction. The author proposed MSA as a stronger, more available and eco-friendly catalyst when compared with other inorganic acids. A LA yield of 22% was achieved at 180 °C, 0.5M MSA, and 20 min. [102].

In 2010, it has been demonstrated that *Gelidium amansii*, which is considered as the category of red macroalgae, can also be converted into LA. This seaweed is rich in carbohydrate content (glucose, galactose, galactan, etc.), which is higher than that of lipid, protein, etc. The authors investigated different reaction conditions and found that LA can be produced at a long reaction time, high reaction temperature, and high catalyst concentration. Hence, by using H_2_SO_4_ as the catalyst with concentration of 3.0%, 9.7 g/L of LA was formed at 160 °C in 43.1 min. [103]. In 2013, another group of researchers worked on the same biomass for LA formation through two steps hydrolysis process. In the first step, they aimed to optimize the hydrolysis condition to produce higher yield of galactose in the liquor and glucose content in the residue. The optimum condition was selected H_2_SO_4_ concentration of 8.97%, temperature of 76 °C, and reaction time of 49 h. The authors believed that first step pretreatment could be a crucial and viable process for hydrolysis of polysaccharides, such as cellulose into monomers, such as glucose. The second step hydrolysis was performed on higher temperature by using the diluted liquor of first hydrolysis step. The best yield of 43% LA was achieved at 180 °C, 3% H_2_SO_4_ concentration, and 48 min. [104].

*Enteromorpha intestinalis* as a green macroalgae were proceeded into fermentable sugars and chemicals. After trying several reaction conditions and their effect on products yield, the authors found that LA could be produced at high temperature, high catalyst concentration, and middling reaction time, although the LA yield was not remarkable. The best LA yield of 4% was achieved with H_2_SO_4_ concentration of 3.7% at 175 °C in 35 min. reaction time [105]. In addition, a group of authors applied another type of green duckweed, *Lemna minor*, with high starch content of 50%, as the raw material to value-added chemicals production process. First, the process of Duckweed cultivation and induction was performed with the uniconazole-induction method. Subsequently, the cultivated duckweed was proceeded to LA in diluted HCl aqueous solution in teflon lined stainless steel autoclave. With the enhancement of HCl concentration, the conversion of the generated glucose into LA and formic acid became faster. The maximum yield of 262 g/kg LA was formed at optimum condition of 1.2 w% HCl, 180 °C and 150 min. [106]. 

As can be seen in the Table 3, among different types of macroalgae, red algae have shown to be a better potential on chemicals production, due to the higher content of polysaccharide complexes in the structure. Although homogeneous catalysts are not promising because of limited recyclability, waste generation, and reactor corrosion, all of the studies have been reported on the homogeneous catalytic hydrolysis of third generation biomass into LA. No published data on using heterogeneous catalyst is available. Therefore, the selection of an appropriate heterogeneous catalyst, to be more stable, efficient, green, and recyclable could play a vital role. 

## 5. Conclusions

LA, as an essential chemical building block, can be directly produced from three generation of biomasses. The acid catalyzed reaction pathway of LA production from biomass could be: (i) pretreatment of biomass, (ii) hydrolysis of polysaccharides to monosaccharides, such as hexoses and pentoses, and (iii) conversion of monosaccharides to LA through several step reactions that are dependent on the types of the sugar. Moreover, there are some unavoidable byproducts when the reaction is catalyzed by acid catalyst in which the type of byproducts depends on the type of biomass, catalyst, solvent, and reaction condition. Byproducts can have some negative effects on the hydrolysis efficiency. Three significant outlooks are listed below on the basis of what was discussed through this review:

(1) Pretreatment of biomass seems to be a compulsory step for improving the yield of LA and reaction rate [32]. Some of the studies performed pretreatment before starting real hydrolysis reaction. There are different methods for biomass pretreatment, which depend on the type of the raw starting compounds. Dilute acid pretreatment was demonstrated as the most common method, especially for second generation biomass. Pretreatment causes an increase in cellulose percentage in the feedstock, and most of the glucose could remain in the pretreated feedstock. Therefore, pretreatment could act as a desirable economic method, which leads to higher efficient LA production. According to the type of biomass, it could be more utile if other types of pretreatment, such as mechanical communication, steam explosion, CO_2_ explosion, pyrolysis, organosolv, and biological pretreatment processes are taken into the account in order to remove some destructive components, such as lignin and inorganic salts [107].
(2) Water seems to be a preferable solvent for the LA production from biomass. Water or supercritical water in the reaction condition is safe and environmental friendly. In addition, it has lots of advantages, such as high thermal conductivity and low viscosity. On the other hand, in some cases, water cannot be an appropriate solvent, due to the insolubility of polymeric feedstock, especially when heterogeneous catalyst is used, limited mass transfer and instability of some water-sensitive catalysts, such as metal chloride [108]. Therefore, using the most suitable ionic and organic solvents could be critical. In recent years, study on ionic liquids has attracted great attention, owing their wide performance as solvents as well as catalysts. However, the harmful effect of this class of solvent, such as toxicity, explosivity, biodegradability, and their high cost limits them from plentiful use [109].
(3) According to what was reported in the recent year literatures, homogeneous catalysts, especially HCl and H_2_SO_4_, were widely used for the conversion of all three generation biomasses. Homogeneous catalysts may be recovered from the reaction solution by using the distillation method, but the challenge of reactor corrosion makes the process outrageously expensive. Replacing homogeneous acid catalysts with green and efficient heterogeneous catalysts can be useful for hydrolysis process in the future. In addition, in recent years, using heterogeneous catalyst in LA production especially from second generation biomass, has gradually increased. Normally, solid catalysts are tunable in the aspect of acidity and reaction condition and they could be easily recovered [110]. Furthermore, heterogeneous catalysts do not have the problem of reactor corrosion and they could be a promising catalyst for industrial use [111]. However, using heterogeneous catalysts still have some limitations, such as limited mass transfer and the deposit of some solid byproducts, such as humins and big organic components on the surface of the catalyst and deactivation over a long period of time.

Furthermore, four outlooks for future trends are highlighted below:

(1) Development of the green heterogeneous catalysts focusing on the important factors, such as surface area, pore size and structure, accessibility of acid sites, recovery and recyclability, and lifetime is the trend for future biomass direct hydrolysis.
(2) Using some novel non-terrestrial resources, such as macroalgae, can demonstrate an important achievement. Therefore, further study is still compulsory for developing an environmental-friendly process with new high recyclable catalysts, which increase the LA yield and progressively target towards raw and cheap biomass feedstock, and finally the possibility to scale up the process going beyond the economic and technological barriers.
(3) Separation and purification of LA from reaction solution, especially while using organic solvent is still a challenge for having a cost-effective application. One potential way to solve this problem is producing a higher LA concentration in the product stream, which can decrease the amount of waste-solvent and reduce the consumption of energy. Moreover, solid acid catalysts are preferred in the separation process. However, work on this research area is still needed.
(4) Formation of by-products, such as thermal-table humins, is still a bottleneck for the industrial scale production of LA. This problem is more relevant while using lignocellulosic biomass (owing to the presence of lignin) as the feedstock. Since humins are prone to blocking and deactivating the catalyst active sites, especially for heterogeneous catalysts, it can limit the scale-up on larger scale. Performing reaction at low temperature, high acid concentration, and using low biomass concentration could be some possible ways of preventing the formation of humins. However, more studies are still needed in order to completely inhibit the formation of humins. In addition, conversion of humins to some new carbon components is suggested for future work.

## Figures and Tables

**Table 1:** Valorization of first generation biomass into LA over catalysts.

Biomass	Solvent	Homo Cat	Hetero Cat	Temperature (°C)	Time (min)	Other	Yield of LA	Ref
**Normal corn starch**	H_2_O	HCl	-	165	15	mw	53–55%	[38]
**High-amylose corn starch**	H_2_O	HCl	-	165	15	mw	53–55%	[38]
**Waxy corn starch**	H_2_O	HCl	-	165	15	mw	53–55%	[38]
**kernel sorghum grain**	H_2_O	H_2_SO_4_	-	200	40		33%	[40]
**Sugar cane molasses**	H_2_O + LA	H_2_SO_4_	-	180	180		30%	[41]
**Sugar beet molasses**	-	-	Amberlyst-36TM	140	180	PRET	78 mol%	[42]

Homo Cat = Homogenous Catalyst; Hetero Cat = Heterogeneous Catalyst; mw = microwave; PRET: PRETREATMENT.

**Table 2:** Valorization of second generation biomass into LA over catalysts.

Biomass	Solvent	Ionic Liquid	Homo Cat	Hetero Cat	T (°C)	Time (min)	Other	Yield of LA	Ref
**Rice straw**	H_2_O	[C_3_SO_3_Hmim]HSO_4_	-	-	180	30		21%	[56]
**Rice straw**	H_2_O		-	S_2_O_8_^2−^/ZrO^2^–SiO_2_–Sm_2_O_3_	200	10		22%	[57]
**Rice straw**	H_2_O		-	S_2_O_8_^2−^/ZrO^2–^SiO_2_–Sm_2_O_3_	200	15	ENZ PRET	25%	[58]
**Rice straw**	H_2_O		-	GaHPMo	175	360		46%	[59]
**Rice straw**	H_2_O + THF + DMSO		HCl	-	180	120	PRET H_2_SO_4_	21%	[60]
**Rice straw**	H_2_O + DCM		HCl, FA, LA	-	200	180		16.6%	[61]
**Paddy Straw**	H_2_O		HCl	-	220	45		24%	[62]
**Cotton straw**	H_2_O		H_2_SO_4_	-	180	60		9.5%	[63]
**Barley straw**	H_2_O		H_2_SO_4_	-	158	15		0.03 g/L	[64]
**Bamboo**	H_2_O	[C_4_(Mim)_2_][(2HSO_4_)(H_2_SO_4_)_4_]	-	-	100	60		47.5%	[65]
**Eucalyptus wood chips**	H_2_O + MeOH		H_2_SO_4_	-	180	90		66 mol%	[25]
**Eucalyptus wood**	H_2_O		H_2_SO_4_	-	170	300	PRET	105 g/L	[53]
**Pinus pinaster wood**	H_2_O		H_2_SO_4_	-	135	600		66%	[67]
**Poplar wood chips**	H_2_O		H_2_SO_4_	-	190	50		17.8%	[68]
**Aspen, pine, fir, birch wood**	H_2_O		H_2_SO_4_	-	220	120		24%	[69]
**Corn stalk**	H_2_O		FeCl_3_	-	230	10		48.7%	[70]
**Corn Stover**	H_2_O	[BMIMSO_3_H] HSO_4_	-	-	95	60		70%	[71]
**Corncob residues**	H_2_O		H_2_SO_4_	-	180	50	7StageH	107.9 g/L	[72]
**Corncob residues**	H_2_O		SnCl_4_	-	180	60		64.6 mol%	[73]
**Corncob**	H_2_O		-	Acid modified zeolite	200	60		52.48ppm	[74]
**Sugarcane bagasse**	H_2_O		H_2_SO_4_	-	150	360		63 mol%	[75]
**Sugarcane bagasse**	H_2_O		H_2_SO_4_	-	170	75	PRET H_2_SO_4_ NaOH	55.00 ± 0.36%	[76]
***Quercus mongolica***	H_2_O		H_2_SO_4_	-	200	10	PRET H_2_SO_4_	16.5% (g/100 g biomass)	[77]
***Quercus mongolica***	H_2_O		-	modified zeolite Y	190	180	PRET H_2_SO_4_	4.6%	[78]
**Empty fruit bunch**	H_2_O		-	hybrid of HY zeolite and CrCl_3_	145	146		53%	[79]
**Kenaf**	H_2_O		-	hybrid of HY zeolite and CrCl_3_	145.2	146.7		66%	[79]
**Oil palm fronds**	H_2_O	[SMIM][FeCl_4_]	-	-	154.5	222		25%	[80]
**Oil palm empty fruit bunch**	H_2_O	InCl_3_^−^[HMIM][HSO_4_]	-	-	160	300		12%	[81]
**Mesocarp fiber**	H_2_O	InCl_3_^−^[HMIM][HSO_4_]	-	-	160	300		13%	[81]
**Oil palm empty fruit bunch**	H_2_O	InCl_3_^−^[HMIM][HSO_4_]	-	-	177	288		17.7%	[82]
**Mesocarp fiber**	H_2_O	InCl_3_^−^[HMIM][HSO_4_]	-	-	177	288		18.4%	[82]
**Giant reed**	H_2_O		HCl	-	180	60	PRET	23%	[83]
**Giant reed**	H_2_O		HCl	-	180	20	mw	21%	[84]
**Carbohydrate-rich potato peel waste**	H_2_O		H_2_SO_4_	CrCl_3_ orAlCl_3_	180	15	mw	49%	[85]
**Fungus *Cortinarius armillatus***	H_2_O		H_2_SO_4_	CrCl_3_ orAlCl_3_	180	40	mw	62%	[85]
**Bamboo shoot shell**	H_2_O	[C_4_mim] HSO_4_	-	-	145	104		71 mol%	[86]

Homo Cat = Homogenous Catalyst; Hetero Cat = Heterogeneous Catalyst; PRET = PRETREATMENT; ENZ = ENZYMATIC; 7StageH = 7 Stage HYDROLYSIS; mw = microwaves.

**Table 3:** Valorization of third generation biomass into LA over catalysts.

Biomass	Solvent	Homogeneous Catalyst	T (°C)	Time (min)	Other	Yield of LA	Ref
***Gracilaria lemaneiformis***	H_2_O	H_2_SO_4_	180	20	mw	16.3%	[99]
***Gracilaria fisheri***	H_2_O	H_2_SO_4_	95	150		3.66 g L^−1^	[100]
***Gracilaria. tenuistipitata***	H_2_O	H_2_SO_4_	95	150		6.12 g L^−1^	[100]
***Gracilaria verrucosa***	H_2_O	H_2_SO_4_	180.9	50		19%	[101]
***Gracilaria verrucosa***	H_2_O	MSA (methanesulfonic acid)	180	20		22%	[102]
***Gelidium amansii***	H_2_O	H_2_SO_4_	160	43.1		9.7 g/L	[103]
***Gelidium amansii***	H_2_O	H_2_SO_4_	180	48	PRET	43%	[104]
***Enteromorpha intestinalis***	H_2_O	H_2_SO_4_	175	35		4%	[105]
***Lemna minor***	H_2_O	HCl	180	150		262 g/kg	[106]

PRET = PRETREATMENT; mw = microwaves.
